# Live birth after fresh embryo transfer vs elective embryo cryopreservation/frozen embryo transfer in women with polycystic ovary syndrome undergoing IVF (FreFro-PCOS): study protocol for a multicenter, prospective, randomized controlled clinical trial

**DOI:** 10.1186/1745-6215-15-154

**Published:** 2014-05-02

**Authors:** Yuhua Shi, Daimin Wei, Xiaoyan Liang, Yun Sun, Jiayin Liu, Yunxia Cao, Bo Zhang, Richard S Legro, Heping Zhang, Zi-Jiang Chen

**Affiliations:** 1 Center for Reproductive Medicine, Shandong Provincial Hospital Affiliated to Shandong University, Reproductive Medical Hospital affiliated to Shandong University, No. 157 Jing Liu Street, Shizhong district, Jinan 250001, China; 2 Reproductive Medical Center of the Sixth Affiliated Hospital, Sun Yat-Sen University, No. 17 Sogoulin Road, Tianhe district, Guangzhou 510655, China; 3 Reproductive Medical Center of RenJi Hospital, Shanghai Jiaotong University School of Medicine, No. 1630 Dong Fang Road, Shanghai 200127, China; 4 Reproductive Medical Center of Jiangsu Province Hospital, No.300 Guangzhou Road, Nanjing 210029, China; 5 Reproductive Medical Center of the first Affiliated Hospital, Anhui Medical University, No. 218 Ji Xi Road, Hefei 230022, China; 6 Reproductive Medical Center, Guangxi Maternal and Child Health Hospital, No. 225 Xin Yang Road, Nanning 530003, China; 7 Department of Obstetrics and Gynecology, Penn State University College of Medicine, 500 University Drive, Hershey, PA 17033, USA; 8 Department of Biostatistics, Yale University School of Public Health, 60 College Street, New Haven, CT 06510, USA

**Keywords:** Frozen–thawed embryo transfer, *In vitro* fertilization, Live birth, Polycystic ovarian syndrome

## Abstract

**Background:**

Polycystic ovary syndrome (PCOS) patients are at increased risk of pregnancy complications, which may impair pregnancy outcome. Transfer of fresh embryos after superovulation may lead to abnormal implantation and placentation and further increase risk for pregnancy loss and complications. Some preliminary data suggest that elective embryo cryopreservation followed by frozen–thawed embryo transfer into a hormonally primed endometrium could result in a higher clinical pregnancy rate than that achieved by fresh embryo transfer.

**Methods/Design:**

This study is a multicenter, prospective, randomized controlled clinical trial (1:1 treatment ratio of fresh vs. elective frozen embryo transfers).. A total of 1,180 infertile PCOS patients undergoing the first cycle of *in vitro* fertilization (IVF) or intracytoplasmic sperm injection will be enrolled and randomized into two parallel groups. Participants in group A will undergo fresh embryo transfer on day 3 after oocyte retrieval, and participants in group B will undergo elective embryo cryopreservation after oocyte retrieval and frozen–thawed embryo transfer in programmed cycles. The primary outcome is the live birth rate. Our study is powered at 80 to detect an absolute difference of 10 at the significance level of 0.01 based on a two-sided test.

**Discussion:**

We hypothesize that elective embryo cryopreservation and frozen–thawed embryo transfer will reduce the incidence of pregnancy complications and increase the live birth rate in PCOS patients who need IVF to achieve pregnancy.

**Trial registration:**

ClinicalTrials.gov Identifier: NCT01841528

## Background

Polycystic ovary syndrome (PCOS) is the most common cause of anovulatory infertility. Although more oocytes than tubal infertile patients are retrieved from women with PCOS during *in vitro* fertilization (IVF), similar pregnancy rates are achieved compared with tubal infertile patients. Some data suggest that women with PCOS may have increased miscarriage rates [1]. A previously reported meta-analysis [2] demonstrated that PCOS patients are at increased risk for pregnancy complications and neonatal complications, including gestational diabetes, pregnancy-induced hypertension, preeclampsia and preterm birth, all of which may lower the chances of live births. Whether that is attributed to the intrinsic characteristics of PCOS, the underlying infertility, the disturbed steroids level or a combination of these factors, however, is unclear [3]. Effective interventions are needed to improve pregnancy outcomes in PCOS patients.

Additionally, both superovulation and assisted reproductive technologies may carry additional risks for abnormal pregnancies. Multiple studies have reported higher risks of abnormal placentation (such as greater placenta weight, placenta previa and preeclampsia), pregnancy complications (such as gestational hypertension) and obstetric complications (such as preterm delivery, low birth weight and high cesarean section rate) in singleton pregnancies induced by assisted conception compared with spontaneous conception [3-6]. Researchers in several observational studies have demonstrated that pregnancies arising from the transfer of frozen–thawed embryos seem to have better obstetric and perinatal outcomes (such as antepartum hemorrhage, small for gestational age (SGA), low birth weight and perinatal mortality) than those induced by fresh embryo transfer [7-9]. Investigators in two independent studies revealed a statistically significant association between elevated peak serum estradiol (E2) levels on the day of the human chorionic gonadotropin (hCG) trigger and the risks of SGA and preeclampsia in singleton pregnancies conceived via IVF [10,11]. Researchers in a cohort study [12] demonstrated that elective cryopreservation of all embryos and subsequent frozen–thawed embryo transfer reduced the odds of SGA and preeclampsia compared with fresh embryo transfer in patients with elevated peak serum E2 levels (>3,450 pg/ml). We hypothesize that supraphysiological steroid level immediately before embryo implantation may represent an independent contributing factor to the increased risk of these pregnancy and obstetric complications.

Unfortunately, most of the current evidence is based on retrospective and uncontrolled studies in which the results may have been confounded by the “second-best” quality of embryos in frozen–thawed cycles. The primary outcome of most existing studies is clinical pregnancy, which is defined as the detection of a fetal heartbeat at 7 weeks gestation. Furthermore, despite the distinct characteristics of infertile PCOS patients compared with tubal infertile patients, few long-term follow up studies have been focused on PCOS patients undergoing IVF.

## Methods/Design

In this multicenter, prospective, randomized controlled clinical trial (1:1 treatment ratio), live birth rates of fresh embryo transfer vs frozen–thawed embryo transfer are being compared in 1,180 infertile PCOS patients undergoing their first cycle of IVF or intracytoplasmic sperm injection (ICSI). Participants will be enrolled at 14 hospitals across mainland China. This study has been approved by the ethics committees at all study sites (listed in Additional file 1). Informed consent will be obtained from each patient before any study procedure is performed, in accordance with good clinical practice. Reporting of the study results will follow the 2010 revised CONSORT statement [13].

### Participants

#### *Inclusion criteria*

The following are the inclusion criteria:

1. Women diagnosed with PCOS according to Chinese PCOS diagnostic criteria

2. Women who have a ≥1-year history of infertility

3. Women ages ≥20 and <35 years old

4. Women who have at least one of the following indications for IVF or ICSI:

a. Ovulation dysfunction and failure to become pregnant after ovulation induction treatment

a. Tubal factors: unilateral or bilateral tubal obstruction, adhesion, unilateral or bilateral salpingectomy or tubal ligation

a. Male factors: oligoasthenozoospermia or obstructive azoospermia

a. Women who are undergoing their first cycle of IVF or ICSI

a. Women whose retrieved oocytes number more than three

The Chinese PCOS diagnostic criteria are menstrual abnormality and either hyperandrogenism or PCO [14]. Menstrual disorders, including oligomenorrhea, amenorrhea or irregular uterine bleeding, are essential for the diagnosis of PCOS. *Oligomenorrhea* is defined as spontaneous cycle length ≥35 days. *Irregular uterine bleeding* is defined as spontaneous cycle length <21 days. *Amenorrhea* is defined as spontaneous cycle length ≥6 months. Hyperandrogenism is diagnosed on the basis of either hirsutism or hyperandrogenemia. Hirsutism is determined by a modified Ferriman–Gallwey score [15] >6 at the screening examination. *Hyperandrogenemia* will be defined as an elevated total testosterone level according to local laboratory criteria. Local cutoffs will be predetermined by each site prior to study initiation. *Polycystic ovarian syndrome* is defined as either an ovary that contains 12 or more follicles measuring 2 to 9 mm in diameter or increased ovarian volume (>10 cm^3^) [16]. Secondary causes of hyperandrogenism and ovulation dysfunction will be excluded before a PCOS diagnosis is confirmed, such as ovarian interstitial tumor or adrenal tumor, congenital adrenal hyperplasia, hyperprolactinemia and thyroid dysfunction.

#### *Exclusion criteria*

The following are the exclusion criteria:

1. Women who have undergone unilateral oophorectomy

2. Women who have previously been diagnosed with a uterine abnormality such as a malformed uterus (uterus unicornis, septate uterus, duplex uterus or uterus bicomis), adenomyosis, submucous myoma or intrauterine adhesion

3. Women or their partners with an abnormal chromosome karyotype not including chromosome polymorphisms, which mainly refer to the variants in the chromosomal heterochromatin region [17]

4. Women who have experienced recurrent spontaneous abortions (including biochemical pregnancy abortion), defined as three or more previous pregnancy losses

5. Women with medical conditions that contraindicate assisted reproductive technology and/or pregnancy, such as poorly controlled type 1 or type 2 diabetes mellitus; undiagnosed liver disease or dysfunction (based on serum liver enzyme test results); renal disease or abnormal serum renal function; significant anemia; history of deep venous thrombosis, pulmonary embolus or cerebrovascular accident; uncontrolled hypertension or known symptomatic heart disease; history of (or suspected) cervical carcinoma, endometrial carcinoma or breast carcinoma; and undiagnosed vaginal bleeding

6. Women who have developed severe ovarian hyperstimulation syndrome (OHSS) during controlled ovarian hyperstimulation (COH)

7. Women with retrieved oocytes numbering three or less

8. Women who are unable to comply with the study procedures

### Interventions

Qualified patients will be randomized into either of two groups. Group A will undergo a fresh embryo transfer after ovarian stimulation, and group B will have all of their embryos cryopreserved after ovarian stimulation and then undergo a frozen–thawed embryo transfer. All of the participants will receive a standardized gonadotropin-releasing hormone (GnRH) antagonist ovarian stimulation protocol and luteal phase support.

#### *Controlled ovarian hyperstimulation and oocyte retrieval*

The following list describes the COH and oocyte retrieval protocol:

1. *Baseline monitoring and initial dose determination*: Recombinant follicle-stimulating hormone (rFSH, Gonal-f; Merck Serono, Geneva, Switzerland) will begin on day 3 of spontaneous menses or cycle induced by exogenous progesterone or oral contraceptive pills. Baseline pelvic ultrasound and basal hormone levels—that is, serum FSH, luteinizing hormone, E2, progesterone and β-hCG test—will confirm an early follicular phase milieu. The initiation doses will be 112.5 IU/day for patients weighing ≤60 kg and 150 IU/day for patients weighing >60 kg.

2. *Ovarian response monitoring and rFSH dose adjustment*: At day 6 of ovarian stimulation, transvaginal ultrasound scans and serum hormone tests will be performed. rFSH doses will be adjusted according to ovarian response. Afterward, such monitoring will be performed either every other day or every day. Human menopausal gonadotropin (Menopur; Ferring Pharmaceuticals, St-Prex, Switzerland) may be added if necessary [18,19].

3. *GnRH antagonist starting time and dose*: GnRH antagonist (cetrorelix; Merck Serono, Darmstadt, Germany) at a daily dose of 250 μg by subcutaneous injection will begin when at least one follicle is ≥12 mm in mean diameter until the trigger day (including the trigger day).

4. *hCG trigger for final oocyte maturation*: hCG will be administered at a dose of approximately 4,000 to 8,000 IU by intramuscular injection when at least two follicles are ≥18 mm in mean diameter.

5. *Transvaginal ultrasound-guided oocyte retrieval*: Oocyte retrieval will be performed 36 hours after hCG injection.

On the oocyte retrieval day, qualified patients will be randomized. For patients assigned to group A, luteal phase support will be started just after follicular aspiration. For patients assigned to group B, no luteal phase support will be administered.

#### *In vitro fertilization and embryo culture*

The oocytes will be inseminated approximately 4 to 6 hours after follicular aspiration. For the scoring of embryos at day 3, we will employ the Puissant *et al.* criteria, with a focus on the number and regularity of blastomeres as well as the percentage fragmentation [20]. Two embryos of top quality will be marked for the first transfer cycle. For patients assigned to group A, two embryos with top quality will be transferred on day 3, and the surplus embryos will been frozen on day 3 or day 5 according to the routine followed at different sites. For patients assigned to group B, all embryos will be cryopreserved and at least two embryos of top quality must be vitrified on day 3. To ensure that two viable, thawed day 3 embryos will be transferred in subsequent programmed cycles, obtaining one or two extra day 3 frozen embryos will be suggested in case frozen embryos lose their viability after thawing.

#### *Embryo transfer and luteal phase support*

##### 

***Group A*** Participants in group A will receive luteal phase support for 2 weeks with intramuscular progesterone obtained on the oocyte retrieval day. On day 3 of embryo culture, two embryos of top quality will be transferred through a catheter using transabdominal ultrasound guidance (full bladder). After embryo transfer, the patient will lie in bed for 30 minutes. If the patient is pregnant, luteal phase support will continue to 10 weeks gestation.

##### 

***Group B*** On the third day of the second menstrual cycle (either spontaneous or induced by giving progesterone or oral contraceptive pills) after COH and oocyte retrieval, patients in group B will receive oral E2 valerate (E2V) at a dose of 4 mg daily. The E2V dose will remain unchanged for 10 days and then will be increased to approximately 6 to 8 mg/day if endometrial thickness is still less than 8 mm. Intramuscular progesterone will be added when endometrial thickness reaches ≥8 mm. Two frozen–thawed embryos will be transferred on the fourth day after progesterone initiation. The transfer procedure will be the same as that used for the fresh embryo transfer. E2V and progesterone will be continued at an unchanged dose until the day on which serum β-hCG levels are tested. If the patient is pregnant, luteal phase support will continue until 10 weeks gestation.

#### *Pregnancy evaluation*

Serum β-hCG will be measured to determine pregnancy 14 days after embryo transfer. If a biochemical pregnancy has been achieved, a transvaginal ultrasound scan will be obtained 35 days after embryo transfer to evaluate the clinical pregnancy. The ultrasound scan will be repeated at 11 weeks gestation to confirm ongoing pregnancy if a biochemical pregnancy has been achieved.

#### *Follow-up evaluation*

At 12 weeks gestation, the presence of first-trimester pregnancy complications (OHSS, miscarriage, ectopic pregnancy and/or gestational trophoblastic disease) will be evaluated by inspecting medical records and will be recorded by completing the case report form for the first pregnancy follow-up time point.

At 28 weeks gestation, the second-trimester pregnancy complications (prenatal diagnosis test results, abortion, gestational diabetes, preeclampsia/eclampsia, incompetent cervix, premature rupture of membrane and/or placenta abruption) will be followed up by telephone call to complete the second pregnancy follow-up time point.

At 37 weeks gestation, the third-trimester pregnancy complications (preterm labor, placenta abruptio, placenta accreta, placenta previa, preeclampsia/eclampsia, intrauterine growth retardation, premature rupture of membrane and/or abnormality of amniotic fluid) will be followed up by telephone call to complete the third pregnancy follow-up time point.

Participants will be required to notify investigators of the time of delivery. An investigator will be sent to the delivery hospital, and placental and cord blood samples will be collected. The delivery information (gestational age, delivery mode, placenta abnormality and/or delivery complications), infant information (for example, birth weight, birth defect) will be recorded by completing forms designed for this fourth follow-up pregnancy visit. The obstetric medical record will be copied, if possible, for the study chart source documents.

At 6 weeks after delivery, postpartum information regarding complications of the mother (for example, postpartum depression, infection, late postpartum hemorrhage) and the infant (for example, neonatal respiratory distress syndrome, neonatal jaundice, neonatal infection, neonatal death, neonatal hospitalization) will be followed up by telephone call for the fifth and final follow-up time point.

If a live birth is not achieved and there are surplus cryopreserved embryos from the first IVF cycle, we will track the outcomes of all frozen embryo transfers in both groups A and B.

### Randomization

Participants (*N* = 1,180) will be allocated randomly into one of the two study groups at a ratio of 1:1. The allocation sequence will be generated by utilizing the ResMan Research Manager central randomization clinical trial management public platform (http://www.medresman.org/). The randomization will be stratified by study site. On the day of oocyte retrieval, investigators will log information into the web database using user identifications and passwords to obtain the randomization numbers and allocated groups for the eligible patients. Both investigators and participants will be aware of the allocations after oocyte retrieval.

### Outcome measurements

#### *Primary outcome*

The primary study endpoint is *live birth*, defined as delivery of any viable infant ≥28 weeks gestation after the first embryo transfer.

#### *Secondary outcomes*

Secondary efficacy parameters will include biochemical pregnancy, clinical pregnancy, implantation rate and ongoing pregnancy. *Biochemical pregnancy* will be defined as a serum β-hCG level ≥10 IU/L 2 weeks after embryo transfer. *Clinical pregnancy* will be defined as the presence of intrauterine gestation sac at 7 weeks gestation. *Ongoing pregnancy* will be defined as a viable pregnancy at 11 weeks gestation. Another secondary outcome will be cumulative live birth results (with each woman having only one allowable live birth) from the single IVF cycle. The safety endpoints will include OHSS, miscarriage, ectopic pregnancy, pregnancy complication, congenital anomalies and neonatal complications.

### Statistical analysis

#### *Sample size and power calculations*

Investigators in a recent study of Chinese infertile PCOS patients undergoing conventional IVF or ICSI with fresh embryo transfers reported that the live birth rate was 31.2 [21]. Imudia *et al.*, in their cohort study reported a live birth rate of 47.6 in cryothawed embryo transfer cycles [12]. In their randomized trial comparing fresh and frozen–thawed transfer in normal responders (average age = 33 years), Shapiro *et al.* (2011) demonstrated that the clinical pregnancy rate per transfer was 84.0 in the cryopreservation group and 54.7 in the fresh embryo transfer group. They reported that the ongoing pregnancy rates per transfer (at 10 weeks gestation) for these two groups were 78.0 and 50.9, respectively [22]. Shapiro *et al.* also showed that the clinical pregnancy rates per transfer in high responders were 79.6 in the cryopreservation group and 65.4 in the fresh embryo transfer group. They found that the ongoing pregnancy rates were 77.6% in the cryopreservation group and 65.4 in the fresh embryo transfer group [23]. In our present study, we plan to test the primary hypothesis of a difference of 10 in the live birth rate for the two randomization arms. Because all of the participants in this study are under 35 years old, we will assume that frozen–thawed embryo transfer will bring about a live birth rate of 40 and fresh embryo transfer will lead to a live birth rate of 30. A sample size of 530 prospectively enrolled participants in each randomization arm will yield a statistical power of 80 at a significance level of 0.01 to demonstrate an absolute difference in live birth rates of 10 between treatment arms. Taking into consideration a dropout rate of 10 (for example, due to canceled cycles or elective cryopreservation of embryos because of increased risk for OHSS), we will enroll 590 participants into each study group.

For the sample size calculations, the significance level will be set at α = 0.01 and the statistical power will be calculated as 1 - β = 0.80. The live birth rate in group A will be set at 0.30. The live birth rate in group B will set at 0.40. The ratio between groups will be 1:1. The minimum sample size will be 530 for each group, for a total of 1,060 participants. Taking into consideration a dropout rate of 10, we expect to ultimately have a total of 1,180 enrollees, with 590 participants in each group.

#### *Type of analysis*

We will utilize an intent-to-treat approach to examine differences in the live birth rates for first embryo transfer cycle in the two treatment arms in the primary analysis by the Pearson *χ*^2^ test. Safety parameters and secondary efficacy parameters, such as pregnancy rate, OHSS rate and other rates, will be analyzed using the Pearson *χ*^2^ test and logistic regression (if necessary). Cox proportional hazards models and the Kaplan-Meier method will be applied to compare time to pregnancy and cumulative live birth rates.

## Trial status

The study was conceived and designed in 2011. The first participant was randomized in June 2013. We completed recruitment in April 2014, and patient follow-up is ongoing.

## Abbreviations

COH: Controlled ovarian hyperstimulation; E2: Estradiol; FSH: Follicle-stimulating hormone; GnRH: Gonadotropin-releasing hormone; hCG: Human chorionic gonadotropin; ICSI: Intracytoplasmic sperm injection; IVF: *In vitro* fertilization; OHSS: Ovarian hyperstimulation syndrome; PCOS: Polycystic ovarian syndrome; SGA: Small for gestational age.

## Competing interests

The authors declare that they have no competing interests.

## Authors’ contributions

ZC designed the whole study. YS supervised the whole project and performed data analysis. DW, XL, YS, JL, YC and BZ supervised patient diagnosis and recruitment. YS and DW conducted data analyses and drafted the manuscript. HZ and RL participated in the manuscript writing and contributed to protocol version 6 development, on which this paper is based. All authors critically reviewed the article and approved the final manuscript.

## Supplementary Material

Additional file 1 (Names of IRBs): The additional file is a Word file listing all of the institutional review boards that provided ethical approval for this trial. Click here for file
