# Supplementary material for: Live birth after fresh embryo transfer vs elective embryo cryopreservation/frozen embryo transfer in women with polycystic ovary syndrome undergoing IVF (FreFro-PCOS): study protocol for a multicenter, prospective, randomized controlled clinical trial
Source: Trials. 2014 May 2;15:154. doi: 10.1186/1745-6215-15-154 (PMC4022358; doi:10.1186/1745-6215-15-154)
Supplement: Additional file 1 — (Names of IRBs): The additional file is a Word file listing all of the institutional review boards that provided ethical approval for this trial. [file 1745-6215-15-154-S1.docx]

Names of all ethical bodies that approved this study in centers involved:

1. Institutional review board of Reproductive medicine Hospital affiliated to Shandong University
2. Institutional review board for medical research of Jiangxi Maternal and Child Health Hospital
3. Reproductive medical review board of Guangxi Maternal and Child Health Hospital
4. Institutional review board of Reproductive Medicine Center in Shenyang City
5. Institutional review board of Sir Run Run Shaw Hospital of Zhejiang University
6. Institutional review board of Renmin Hospital of Wuhan University
7. Institutional review board of Jiangsu Province Hospital
8. Institutional review board of the Sixth Affiliated Hospital of Sun Yat-Sen University
9. Institutional review board of the First Affiliated Hospital of Anhui Medical University
10. Institutional review board of Renji Hospital affiliated to Shanghai Jiaotong University School of Medicine
11. Institutional review board of Sun Yat-Sen Memorial Hospital of Sun Yat-Sen University
12. Institutional review board of Yuhuangding Hospital of Yantai
13. Institutional review board of Shanxi Provincial Maternity and Children's Hospital
14. Institutional review board of Suzhou municipal Hospital
